# Genome-Wide Identification and Expression Analysis of the Aux/IAA Gene Family in Lettuce (*Lactuca sativa* L.)

**DOI:** 10.3390/ijms26041687

**Published:** 2025-02-16

**Authors:** Jingqing Zhang, Yikun Xu, Guize Wu, Chaoyong Niu, Yibo Zhang, Jinghong Hao, Baoju Wang, Ning Liu

**Affiliations:** 1 Beijing Vegetable Research Center, Beijing Academy of Agriculture and Forestry Sciences, Beijing 100097, China; 2 National Engineering Research Center for Vegeta-bles, Beijing Academy of Agriculture and Forestry Sciences, Beijing 100097, China; 3 Beijing Key Laboratory of New Technology in Agricultural Application, National Demonstration Center for Experimental Plant Production Education, Plant Science and Technology College, Beijing University of Agriculture, Beijing 102206, China; 4 State Key Laboratory of Vegetable Biobreeding, State Key Laboratory of Urban Agriculture (Northern China), Beijing Academy of Agriculture and Forestry Sciences, Beijing 100097, China; 5 College of Food Science and Engineering, Shandong Agricultural University, Tai’an 270108, China

**Keywords:** Aux/IAA, auxin response, lettuce, bolting, high temperatures

## Abstract

The Aux/IAA proteins are key regulators of auxin signaling transduction, mediating various physiological and developmental processes in higher plants; however, little information on Aux/IAAs is known in lettuce, an economically important vegetable. In this study, a total of 29 *LsAux/IAA* genes were identified from the lettuce genome. Sequence alignment and domain analyses suggested the presence of conserved Aux/IAA subdomains (Domain I-IV) in those LsIAAs, and Phylogenetic analysis indicated that members of LsAux/IAA could be classified into 10 subgroups by their homology to *Arabidopsis* Aux/IAAs, which is also supported by exon-intron structure, consensus motifs, and domain compositions. Transcriptome data suggested that most of the *LsIAA* genes were expressed in all tissues, whereas some of them were preferentially expressed in specific tissues. Analysis of cis-acting elements indicated the *LsIAA* genes might be regulated by hormonal treatments and abiotic stresses, which was confirmed by qRT-PCR experiments. Taken together, our study provides valuable information for further investigation of the biological roles of *LsIAA* genes in high-temperature conditions.

## 1. Introduction

Auxin, a pivotal phytohormone, orchestrates diverse aspects of plant growth, development, and responses to environmental stresses [1,2,3]. The canonical auxin signaling pathway is primarily mediated by three core components: the TIR1 (Transport Inhibitor Response 1) F-box protein, the Aux/IAA (Auxin/Indole-3-Acetic Acid) family of transcriptional repressors, and the ARF (Auxin Response Factor) family of transcription factors. The signaling cascade is initiated when auxin binds to the TIR1 receptor, facilitating the formation of the SCFTIR1 ubiquitin ligase complex [4,5]. This complex subsequently targets Aux/IAA proteins for ubiquitination and degradation, thereby releasing ARFs from their repressive Aux/IAA complexes [6]. The liberated ARFs can then bind to Auxin Response Elements (AuxREs) in the promoters of auxin-responsive genes, leading to their transcriptional activation [7]. Thus, the auxin signaling pathway is intricately regulated by Aux/IAA transcriptional repressors, which are central to the transduction of auxin signals.

Aux/IAA proteins are characterized by four highly conserved domains (I–IV), which underpin their functional properties [8,9]. Domain I, located at the N-terminus, contains an “LxLxL” motif, also known as the ethylene response factor (ERF)-associated amphiphilic repression (EAR) motif, responsible for transcriptional repression [7]. Domain II harbors the “GWPPV” motif, a degron that regulates the turnover of Aux/IAA proteins upon auxin perception [10]. Between domains I and II, a conserved “KR” motif and a bipartite nuclear localization signal (NLS) are essential for nuclear localization [11]. At the C-terminus, domains III and IV together form a type I/II Phox and Bem1p (PB1) domain, which facilitates dimerization with other Aux/IAA or ARF proteins [12,13]. However, structural diversity has been reported in some Aux/IAA proteins, with the absence of certain domains leading to non-canonical auxin signaling pathways, particularly under environmental stresses [14]. For instance, mutations in domain II can render Aux/IAA proteins resistant to auxin-induced ubiquitination, enabling specific auxin responses [15].

To date, the *Aux/IAA* gene family has been extensively studied in model plants such as *Arabidopsis thaliana*, as well as in several vegetables [8]. In *Arabidopsis*, 29 *Aux/IAA* genes have been identified, with accumulating evidence highlighting their critical roles in plant growth and development [8]. For example, AtIAA8 regulates flower organ development by modulating jasmonic acid (JA) levels through interactions with AtARF6/8, while AtIAA12 controls root meristem formation during early embryogenesis by interacting with AtARF5 [16]. Additionally, AtIAA12 forms heterodimers with AtARF4, inhibiting its interaction with AtARF5 to regulate stem regeneration [17]. Beyond developmental roles, Aux/IAA proteins are implicated in stress responses. For instance, AtIAA5 and AtIAA19 expression is directly modulated by DREB transcription factors, and mutations in these genes enhance drought sensitivity [18]. Similar observations were reported in apples (*Malus pumila* Mill.). Transgenic apple calli overexpressing *MdIAA8/9/25* exhibited higher tolerance to salt stress, and expression of some salt-responsive genes was increased accordingly [19]. Thus, Aux/IAA repressors serve as critical nodes integrating signals from both abiotic stress and developmental pathways.

Lettuce (*Lactuca sativa* L.), a member of the Asteraceae family, is a globally important leafy vegetable [20,21]. A notable feature of lettuce is its rapid transition from vegetative to reproductive growth under warm temperatures, accompanied by increased auxin levels in the shoot apex, stem elongation, and early flowering [22]. As the primary edible organs of lettuce are leaves and stems, thermally induced bolting poses a significant challenge for lettuce breeding [23,24]. Auxin and ARFs have been shown to play major roles in this transition process [25], leading us to hypothesize that Aux/IAA repressors are crucial regulators of lettuce bolting under warm temperatures [22,26]. Despite the release of the annotated lettuce genome in 2017 [21], a comprehensive genome-wide analysis of the lettuce *Aux/IAA* gene family has not been investigated yet. In this study, we performed a genome-wide analysis of the *Aux/IAA* gene family in lettuce, identifying 29 *LsIAA* genes. We systematically characterized their physical and chemical properties, genomic structures, chromosomal locations, conserved domains, cis-elements, evolutionary relationships, expression profiles, and subcellular localizations. Our findings provide a foundational molecular framework for the *LsIAA* gene family, paving the way for future functional characterization of *LsIAA* genes in lettuce growth, development, and stress responses.

## 2. Results

### 2.1. Identification of IAA Members in Lettuce

To explore *IAA* candidate genes in the lettuce genome, the *Arabidopsis IAA* genes were used as the query sequences. Then, a total of 29 *LsIAA* genes were obtained through domain identification and the removal of redundant sequences. Based on their locations on the chromosome, we renamed these genes *LsIAA1-LsIAA29*. All LsIAA protein sequences are displayed in Supplementary Table S1. The physical and chemical properties of *LsIAAs* are as follows (Table 1). The proteins encoded by *LsIAAs* range from 97 amino acids (*LsIAA20*) to 340 amino acids (*LsIAA11*). Their relative molecular mass also varies, ranging from 11.00 kDa to 35.05 kDa. The theoretical isoelectric points (pI) of LsIAAs ranged from 4.64 to 8.54, which may also indicate that LsIAAs can participate in physiological and biochemical reactions in different environments. From the point of view of the instability index (II) of these proteins, seven proteins (LsIAA3/4/6/9/13/17/27) have an instability coefficient below 40, which indicates that they may exist stably in vitro. The aliphatic index (AI) is also a standard to measure the stability of proteins, and the higher the value of proteins, the more stable they are. The AI of LsIAA4/16/17, which was above 80, was the highest among all LsIAA proteins.

The locus number of *LsIAAs* is provided according to the Phytozome. MW indicates theoretical molecular weight; pI, theoretical isoelectric point; II, instability index; AI, aliphatic index.

### 2.2. Phylogenetic Analysis of IAAs

**Figure 1:**
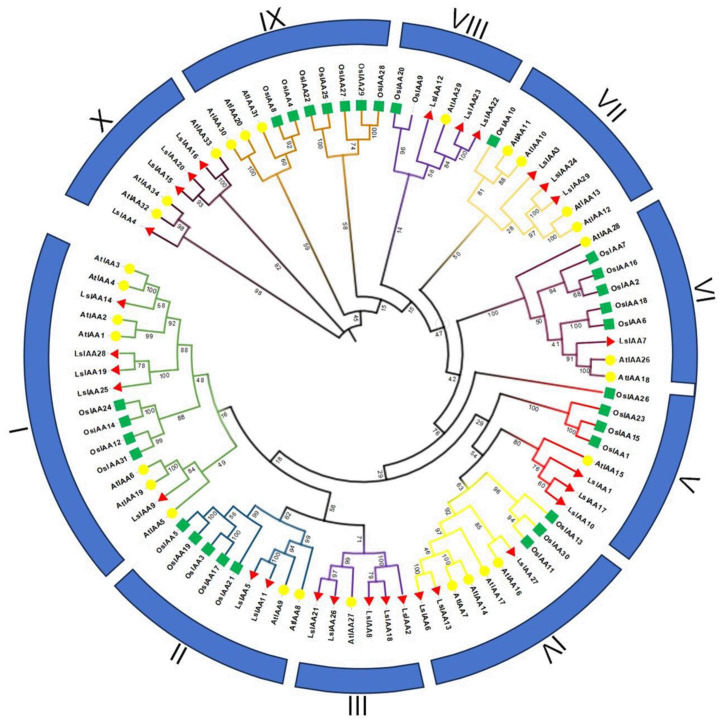
Phylogenetic tree of IAA proteins among *Arabidopsis*, rice, and lettuce. IAA proteins were divided into 10 groups (I–X). The different shapes and colors of the symbols indicate different species. The red triangle indicates *Lactuca sativa*, the yellow circle indicates *Arabidopsis thaliana*, and the green square indicates *Oryza sativa*.

To explore their evolutionary relationship, all IAA proteins in *Arabidopsis* (29), rice (31), and lettuce (29) were aligned to generate a phylogenetic tree (Figure 1). Based on their relationships in the phylogenetic tree, the 89 IAA proteins were categorized into 10 groups (I–X). IAA proteins of all three species were present in all groups, except for the absence of OsIAA proteins in group III/X and LsIAA proteins in group IX. Group I had the most IAA proteins, with sixteen from all three species, whereas group III/VIII only had six proteins. In the phylogenetic tree, each LsIAA was identified as one or more homologs of AtIAA, suggesting they may have the same function in plants. In addition, the phylogenetic tree showed that Aux/IAA members in lettuce were closely related to those in *Arabidopsis*.

### 2.3. Analysis of Gene Structure and Conserved Domain

**Figure 2:**
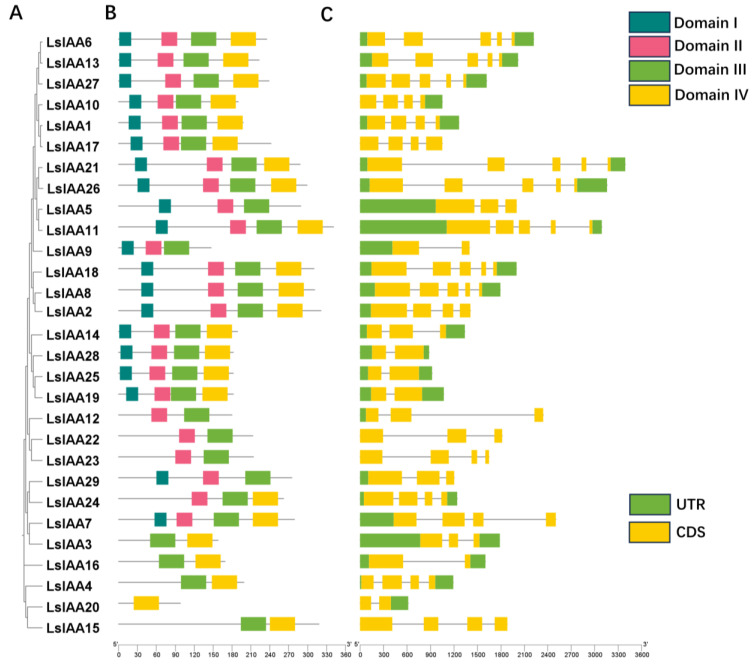
Analysis of conserved domains and gene structure of *LsIAAs*. (**A**) *LsIAA* gene family phylogenetic tree. (**B**) Conservative domain analysis. Domain I-IV are shown in blue, pink, green, and yellow, respectively. (**C**) The UTRs region, exons, and introns are indicated by green boxes, yellow boxes, and black lines. The ruler at the bottom measures the length of the exon and intron.

In general, Aux/IAAs contained four conserved domains (Domain I-IV), and the vast majority of LsIAA proteins (17) contained these four conserved domains (Figure 2). There were nine members (LsIAA3/4/12/15/16/20/22/23/24) missing domain I, suggesting that the transcription inhibition function of these proteins may be impaired. The absence of domain II (LsIAA3/4/15/16/20) made them more stable and difficult to degrade. Several proteins (LsIAA5/9/12/22/29) contained the structure of the truncated domain IV c-terminal, indicating those LsIAA proteins were less likely to form dimers. Only LsIAA20 was missing domain III, which may also result in the inability to form dimers with LsARF as well as the absence of domain IV.

The distributions of introns and exons were used to analyze the gene structure of *LsIAAs*. All the exons of *LsIAAs* are in the range of 2–5. Interestingly, it is noteworthy that *LsIAA* genes on the same branch have the same intron-exon structure, indicating that they may be highly conserved in the same group. Similarly, the distribution of conserved domains was highly consistent, suggesting that these genes located on the same branch were highly conserved.

### 2.4. Chromosomal Location and Synteny Analysis of LsIAA Genes

**Figure 3:**
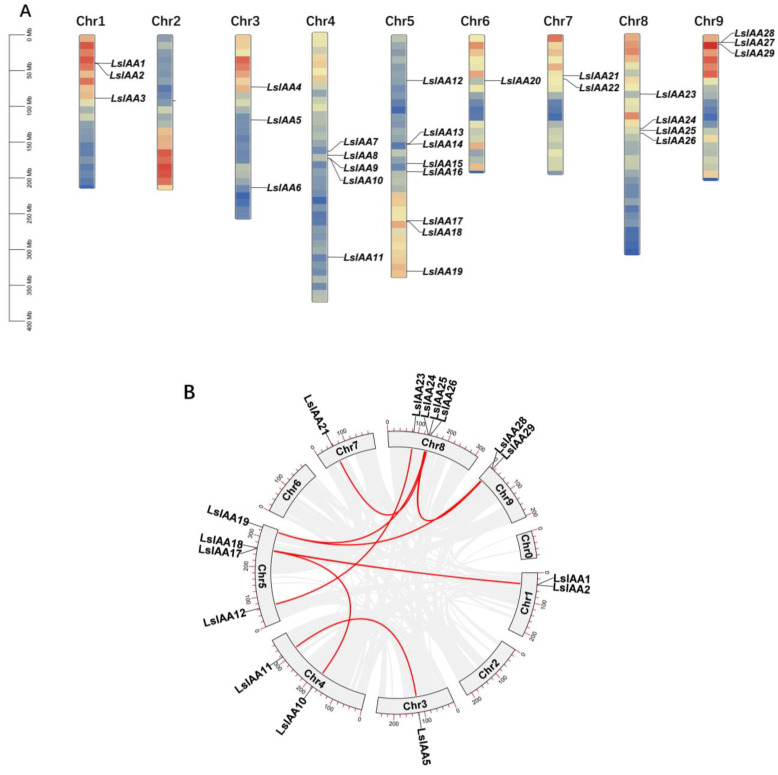
Chromosomal location and collinearity analysis of *LsIAAs*. (**A**) The chromosome number is indicated at the top, and the scale on the left represents the chromosome size. (**B**) Collinearity analysis of *LsIAAs*. The gray line in the background indicates a collinear relationship in the whole lettuce genome, and the red line represents the collinear relationship among *LsIAAs*.

The results showed that a total of 29 *LsIAAs* genes were unevenly distributed on the eight chromosomes of lettuce (except chromosome 2) (Figure 3A). Chromosome 5 contained the highest number of *LsIAAs* (8) and chromosome 4 contained 5 *LsIAAs*. In contrast, chromosome 6 contained only 1 *LsIAA* gene, and chromosome 7 contained only 2 *LsIAA* genes.

**Figure 4:**
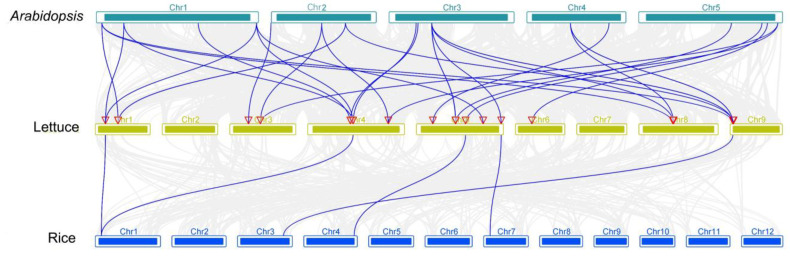
Synteny analysis of *IAA* genes in the genomes of *Arabidopsis*, rice, and lettuce. The gray line in the background indicates Colinear relationships in lettuce and *Arabidopsis* genomes or lettuce and rice genomes, and the blue line represents the collinear relationship among *LsIAAs* in lettuce and *Arabidopsis* genomes or lettuce and rice genomes.

Collinearity diagrams of *LsIAAs* were further analyzed. As shown in Figure 3B, the results revealed that 29 *LsIAA* genes were involved in 12 segmental duplication events. It is worth mentioning that these 12 pairs of fragment duplicated genes were on different chromosomes. In addition, nonsynonymous (Ka) and synonymous (Ks) substitution rates were calculated for each homologous gene pair using the TBtools program (Supplementary Table S2). All *LsIAA* homozygote pairs had Ka/Ks ratios less than 1. Furthermore, 31 homologous pairs of genes were identified between lettuce and *Arabidopsis*, and five homologous pairs of genes were identified between lettuce and rice, respectively (Figure 4). Eight *LsIAAs* (*LsIAA4/12/14/16/17/19/20/24*) in *Arabidopsis* had one homologous gene. In addition, nine *LsIAAs* (*LsIAA1/5/7/11/13/25/27/28/29*) had 2 homologous genes, and two *LsIAAs* (*LsIAA3/10*) had 3 homologous genes. In contrast, only five *LsIAAs* have 1 homolog in rice each, and these five genes also have homologs in *Arabidopsis*. The high homology between lettuce and *Arabidopsis IAAs* indicates that the sequence and function of lettuce are highly conserved and closely related to *Arabidopsis*.

### 2.5. Analysis of Cis-Acting Element in the Promoter Region of LsIAA Genes

**Figure 5:**
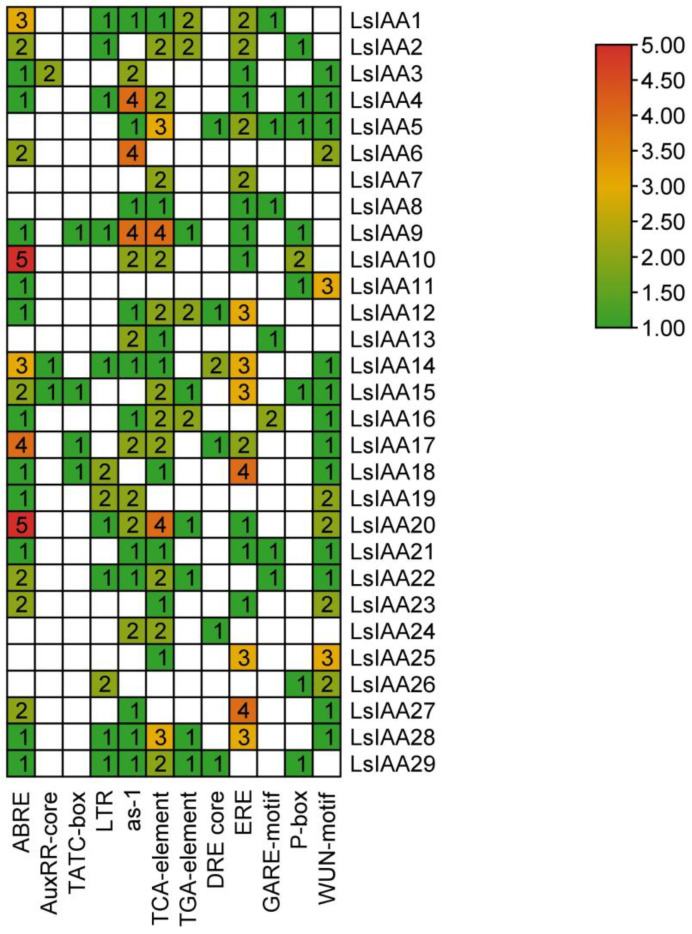
Identification of the cis-acting elements in the promoter of *LsIAAs*. ABRE and DRE core, ABA-responsive element; AuxRR-core and TGA-element, cis-acting element involved in auxin-responsive element; LTR, cis-acting element involved in low-temperature response, TCA-element, cis-acting element involved in SA response; GARE-motif, P-box and TATC-box, cis-acting element involved in GA response.

To identify potential regulation, we analyzed all cis-acting elements 2kb upstream of the *LsIAAs*. All cis-acting elements within 2kb upstream of *LsIAAs* are listed in Supplementary Table S3. Excluding some duplicated cis-acting elements as well as common cis-acting elements (light-responsive elements), we identified a total of 12 types of cis-acting elements totaling 255 major cis-acting elements (Figure 5), with the five most abundant cis-acting elements being the abscisic acid response element (ABRE), TCA-element, ethylene-responsive element (ERE), as-1 and Wun-motif. Among them, ABRE was found in 22 *LsIAA* members and occurred 43 times, suggesting that most of the *LsIAAs* may be involved in the abscisic acid response process. They may play an important role in plant growth and response to adversity. 44 TCA-elements and 37 as-1 were identified in 21 and 23 *LsIAAs*, respectively, which were both involved in the salicylic acid response process and may play an important role in biotic and abiotic stresses such as disease resistance as well as root development in plants. AuxRR-core (4) and TGA-element (14), the auxin response element, were found in 3 and 10 *LsIAAs*, respectively, suggesting that these few *LsIAAs* may play a major role in the auxin signaling pathway.

### 2.6. Expression Patterns of LsIAA in Different Organs

**Figure 6:**
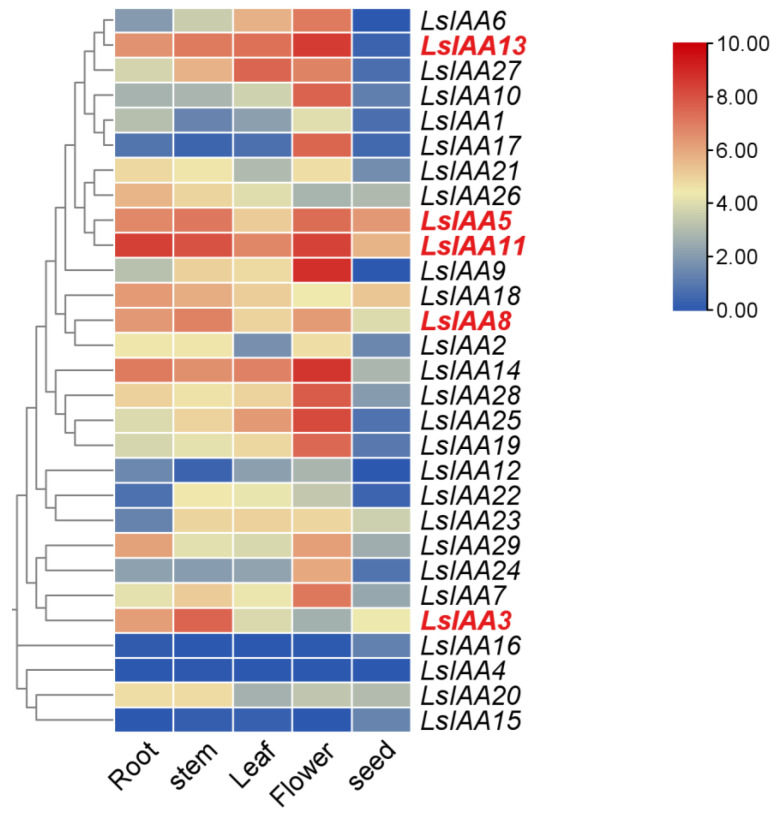
Expression profiles of *LsIAA* genes in different organs and tissues. Expression levels of *LsIAA*s are indicated as the log_2_-based fluorescence intensity values to generate a heatmap by using TBtools. *LsIAA*s with high expression levels in stem were labeled in red.

The temporal and spatial expression of genes may provide insights into their functional characteristics. To determine the tissue-specific expression pattern of *LsIAA* genes in different tissues (root, stem, leaf, flower, and seed), the expression of *LsIAAs* was analyzed based on transcriptomic data in NCBI. As shown in Figure 6, the expression of these genes varied in different tissues. For instance, *LsIAA5/11/13/14* was highly expressed in lettuce, suggesting that they may be involved in the growth and development of various plant tissues. *LsIAA7/9/19/25/28* were highly expressed only in flowers, implying that they may be involved only in the development of the floral organs. Due to the high expression in stems, *LsIAA3/5/8/11/13* may be involved in stem differentiation or even bolting.

### 2.7. Expression Analysis of LsIAAs Under High-Temperature Treatment

**Figure 7:**
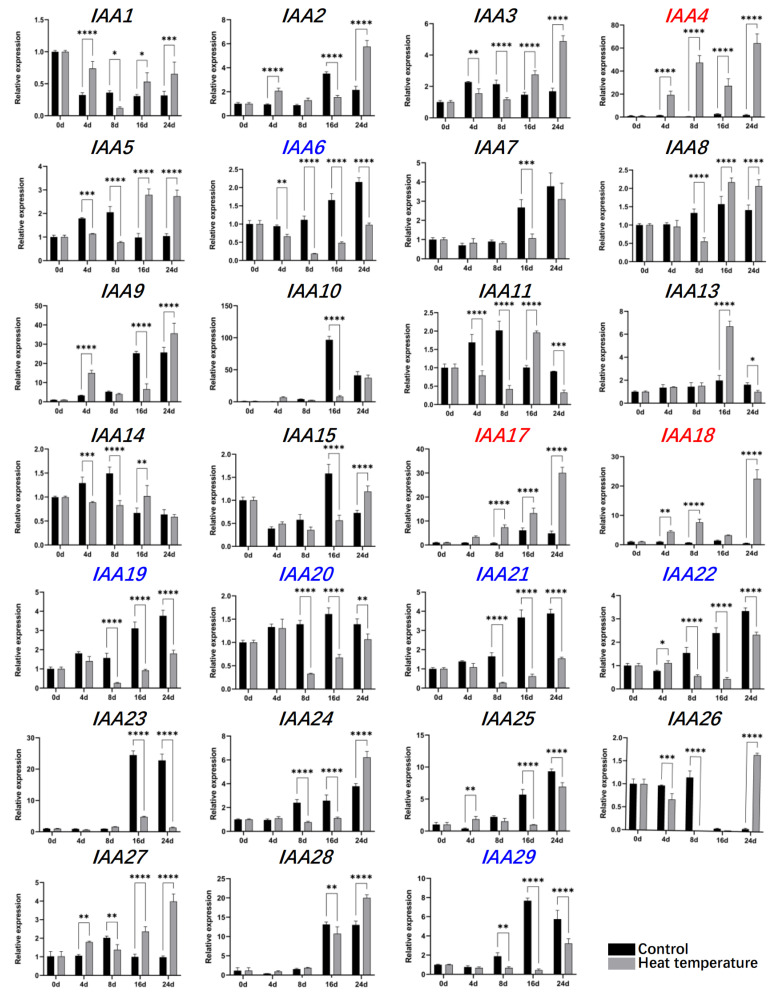
Expression analysis of *LsIAAs* in lettuce under high temperature. The error bars represent the standard error of the means of three independent replicates. Stars above the error bars indicate significant differences between treatments and the controls through unpaired Student’s *t*-test. * stands for *p* < 0.05, ** stands for *p* < 0.01, *** stands for *p* < 0.001, and **** stands for *p* < 0.0001. The *LsIAAs* with significantly upregulated expression levels at high temperatures were labeled in red. The *LsIAAs* with significantly downregulated expression levels over time compared to the control group were labeled in blue.

To explore the potential mechanism in high-temperature bolting, the expression levels of *LsIAAs* were examined in the stem tissues of lettuce. Expression patterns of most *LsIAAs* may be affected by high temperature, due to *LsARFs* being regulated by high temperature (Figure 7). The results showed that the expressions of *LsIAA4/17/18* were significantly up-regulated at high temperatures, which may imply that high temperature induces the expression of these genes. In contrast, the expressions of *LsIAA6/19/20/21/22/29* decreased significantly with time compared to the control, suggesting that these genes may be suppressed by high temperature. A previous study found that lettuce started bolting on the 8th day after the high-temperature treatment, and we hypothesized that bolting affected the expression of these genes [27]. For example, the expressions of *LsIAA3/5/8* were lower than that of the control group before 8 days, but higher than that of the control group after 8 days, which may imply that these genes have different functions after flower bud differentiation.

In addition, there were still expressions of some *LsIAAs* changed over time, without a stable pattern. The transcriptional levels of *LsIAA1/27* were lower than those of the control only on day 8. However, the expression level of *LsIAA13* was significantly up-regulated only on day 16.

### 2.8. Subcellular Localization of LsIAAs

**Figure 8:**
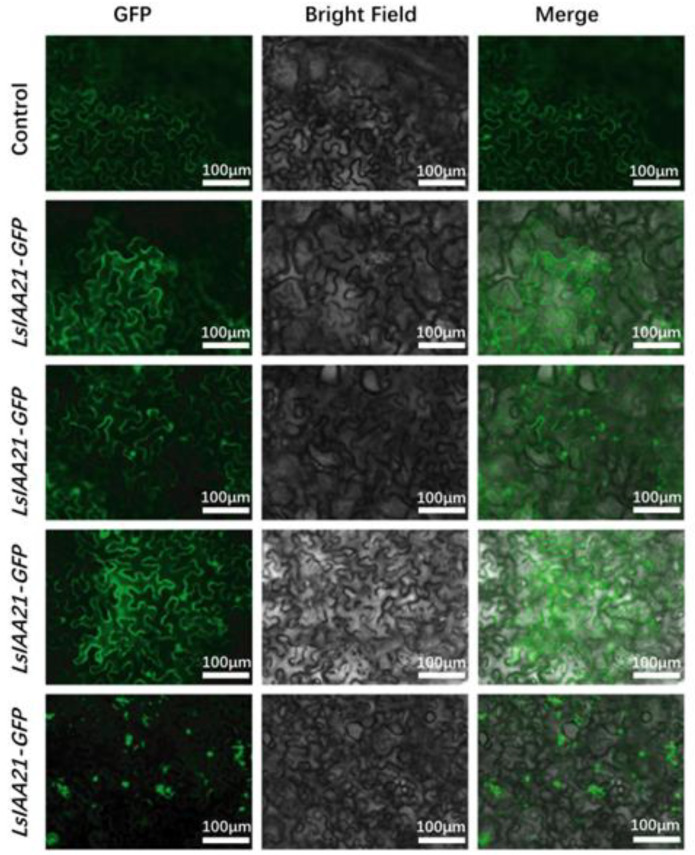
Subcellular localization of transiently expressed *LsIAA3/4/17/21–GFP* and fusion protein in *N. benthamiana* leaves. Scale bars, 100 μm. The subcellular localization assay was repeated three times with similar results.

Based on gene expression analysis, we selected four *LsIAAs* whose expressions were repressed or induced upon high temperatures (Figure 8). The *LsIAA3/4/17/21*-*GFP* construct was generated driven by a 35S promoter and transformed into *N. benthamiana* leaves. The results show that LsIAA3/4/17 proteins were localized to the nucleus and membranes, whereas LsIAA21 protein was localized only in the nucleus.

### 2.9. Predicted LsIAA-LsARF Interaction Networks

**Figure 9:**
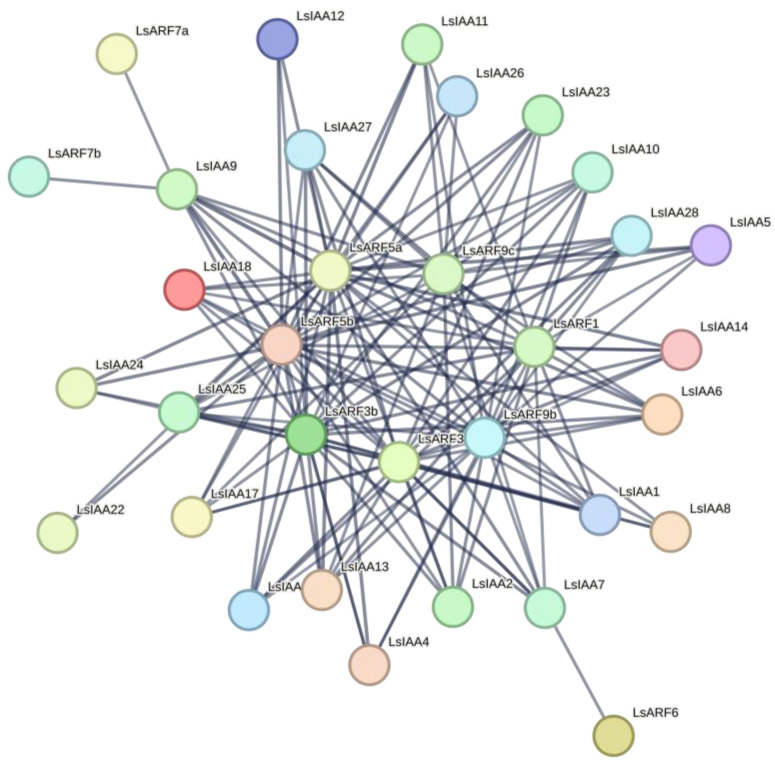
Predicted protein-protein interaction network of LsIAAs and LsARFs. By setting the minimum interaction score at 0.7, 23 LsIAAs and 9 LsARFs formed a protein-protein interaction network.

To better explore the functional networks of LsIAAs and LsARFs, the protein-protein interaction network was predicted by using STRING (Figure 9). By setting the minimum interaction score at 0.7, 23 LsIAAs and 9 LsARFs formed a protein-protein interaction network. The results showed that most LsIAAs can interact with ARFs. It is worth noting that 7 LsARFs (LsARF1/3a/3b/5/5b/9b/9c) may interact strongly with most LsIAA proteins (both canonical and non-canonical). This may also imply that there is competition for LsARF between typical and atypical LsIAA. Interestingly, in this network, LsARF6 only interacted with LsIAA7 and LsARF7a/7b interacted with LsIAA9, which may make it easier to find the auxin signaling pathways of these three *LsARF* genes.

## 3. Discussion

### 3.1. Evolutionary Conservation of Lettuce Aux/IAA Genes

Auxin/indole-3-acetic acid (Aux/IAA) proteins are fundamental components of the auxin signaling pathway, a pivotal regulatory system controlling a wide variety of plant growth and developmental processes [28]. The canonical model of auxin signaling describes Aux/IAA proteins as early responders to fluctuations in cellular auxin concentrations [8,29]. Thus, extensive research on the *Aux/IAA* gene family in model organisms, such as *Arabidopsis thaliana*, has provided valuable insights into the functional roles of these proteins in regulating plant growth and stress responses. Moreover, the application of genome sequencing techniques has facilitated the identification of *Aux/IAA* gene families across various plant species, including Chinese cabbage (*Brassica rapa*), cucumber (*Cucumis sativus*), legume (*Medicago truncatula*), and corn (*Zea mays*), with these species showing a range of 17 to 55 *Aux/IAA* genes [30,31,32,33]. In our study, a comprehensive analysis of the lettuce genome revealed the presence of 29 *Aux/IAA* genes, a number that aligns closely with the number of *Aux/IAA* genes identified in *Arabidopsis* despite a significant disparity in genome size. Lettuce has a genome approximately 14 times larger than *Arabidopsis*, yet the two species exhibit a similar number of *Aux/IAA* genes. This observation suggests that, despite differences in genome size and complexity, the *Aux/IAA* gene family has undergone significant evolutionary pressure to maintain a stable number of functional genes. The conservation of gene numbers across different plant species likely reflects the indispensable role these proteins play in regulating key biological functions. This stability in gene number could be the result of selective pressures that favor the retention of crucial regulatory genes, which are essential for maintaining the functional integrity of the auxin signaling pathway [34,35]. Additionally, the occurrence of multiple whole genome duplication events in lettuce might have prompted the selective retention or functional divergence of *Aux/IAA* genes, ensuring that their core regulatory roles were preserved while avoiding redundant gene copies.

### 3.2. Structural and Functional Diversity of LsIAA Proteins

The characterization of the lettuce Aux/IAA proteins revealed that most of the identified proteins contained four conserved domains, consistent with the canonical structure of Aux/IAA proteins [8]. However, several LsIAA proteins exhibited partial or complete deletions in one or more of these conserved domains, suggesting the existence of non-canonical Aux/IAA proteins in lettuce. Specifically, nine LsIAA proteins were found to lack domain I, which is critical for the transcriptional repression of ARFs. This absence could impair the function of these proteins in the canonical auxin signaling pathway. Furthermore, five LsIAA proteins, including LsIAA15, LsIAA16, and LsIAA20, exhibited deletions in domain II, which is involved in the degradation of these proteins via the ubiquitin-proteasome pathway. These deletions were associated with increased protein stability, as reflected by their high protein instability index values. Similarly, truncated versions of domain IV were observed in LsIAA5, LsIAA9, LsIAA12, LsIAA22, and LsIAA29, which would likely disrupt their ability to form dimers with ARFs or other Aux/IAA proteins. These findings align with previous studies on non-canonical Aux/IAA proteins in other species, such as *Arabidopsis* and tomato, where deletions or modifications in conserved domains resulted in the loss of traditional transcriptional repression activity [36]. The presence of non-canonical Aux/IAA proteins with altered domain structures suggests that these proteins may function through alternative mechanisms. For example, in *Arabidopsis*, non-canonical IAA proteins, such as AtIAA32, have been shown to interact with receptor-like kinases, such as TMK1, or to participate in auxin signaling through distinct regulatory pathways [37]. This flexibility in signaling mechanisms underscores the complexity of the auxin signaling network and its capacity to integrate various developmental and environmental signals. In lettuce, as in other species, the functional divergence of these non-canonical IAA proteins may represent an adaptation to specific physiological needs, offering a broader range of regulatory potential in response to fluctuating environmental conditions.

### 3.3. LsIAAs Are Implicated in Thermal-Induced Bolting in Lettuce

Recent studies have highlighted the involvement of Aux/IAA proteins in plant responses to various abiotic stresses, such as drought, cold, and heat. For example, in rice, overexpression of *OsIAA6* and *OsIAA18* has been shown to enhance drought tolerance by activating abscisic acid (ABA) signaling pathways [38], while silencing of *OsIAA20* results in increased susceptibility to drought [39]. Similarly, *Arabidopsis iaa* mutants, such as the *iaa5/6/19* triple mutant, exhibit reduced drought tolerance, further supporting the role of *Aux/IAAs* in abiotic stress responses [40]. These findings indicate that Aux/IAA proteins serve as key integrators of developmental and stress signaling pathways, coordinating the plant’s response to both environmental cues and internal growth signals. In the context of heat stress, *Arabidopsis iaa11* mutants have been shown to exhibit increased sensitivity to UV-A/B, suggesting that Aux/IAA proteins may also play a role in UV stress tolerance. In rice, heat-responsive proteins like OsIAA29 are critical for seed development, particularly during the grain-filling stage, under high-temperature conditions [41]. Given the established role of auxin in regulating biological processes such as bolting, flowering, and stress responses, it is plausible that Aux/IAA proteins in lettuce might also be involved in heat-induced bolting, a critical developmental transition in this species.

As predicted, we observed that the expression of several *LsIAA* genes was influenced by heat stress, with particular attention given to the expression profiles of *LsIAA3*, *LsIAA5*, *LsIAA8*, and *LsIAA11*. These genes, which are preferentially expressed in stem tissues, showed a significant reduction in expression levels following an 8-day heat treatment, followed by a de-repression of their expression during prolonged heat exposure. This dynamic regulation of *LsIAA* expression under heat stress conditions could play a role in the heat-induced bolting process in lettuce. Our previous studies have demonstrated that heat treatments can trigger bolting in the GB-30 lettuce cultivar, with the upregulation of certain *LsARF*s, such as *LsARF3*, *LsARF5*, and *LsARF8a*, promoting this developmental transition [22,23]. It is conceivable that the downregulation of *LsIAA3*, *LsIAA5*, *LsIAA8*, and *LsIAA11* under heat stress may facilitate the activation of LsARF3- and LsARF5-mediated gene expression, thereby driving the bolting process. To further elucidate the role of LsIAAs in heat-induced bolting, future studies should focus on investigating the direct interactions between LsIAAs and LsARFs. Understanding how LsIAAs modulate the activity of ARFs under heat stress will provide valuable insights into the molecular mechanisms governing bolting in lettuce and other temperature-sensitive plants.

## 4. Materials and Methods

### 4.1. Identification of LsIAA Genes from Lettuce Genome

To identify members of the *Aux/IAA* genes in lettuce, full-length protein sequences of 29 *IAA* gene family members of *Arabidopsis* were obtained by downloading from the *Arabidopsis* public database TAIR (https://www.arabidopsis.org, accessed on 1 July 2024) and used as query sequences for BLASTp search in Phytozome v13 (https://phytozome-next.jgi.doe.gov/, accessed on 1 July 2024) with default parameters to obtain all IAA candidate proteins of lettuce. Subsequently, the sequences of the candidate members were analyzed using CDD-search (https://www.ncbi.nlm.nih.gov/Structure/cdd/cdd.shtml, accessed on 1 July 2024) to exclude those that did not contain the conserved structural domain of Aux/IAA (PF02309), and the redundant members were removed by multiple sequence matching, and the ones obtained after screening were the members of the *LsIAA* gene family. The amino acid number, isoelectric point, molecular weight, instability index, and aliphatic index of the LsIAA proteins were predicted by using the ProParam tool (https://web.expasy.org/protparam/, accessed on 1 July 2024).

### 4.2. Sequence Alignment and Phylogenetic Construction

The sequences of LsIAA proteins and AtIAA proteins were compared in multiple sequences using ClustalW, and a phylogenetic tree was constructed by using the neighbor-joining method in MEGA-X software with default settings. Bootstrap was set to 1000 times.

### 4.3. Analysis of Characterization, Gene Structure, and Conserved Domain of LsIAA Genes

The amino acid number, isoelectric point, molecular weight, instability index, and aliphatic index of the LsIAA proteins were predicted by using the ProParam tool (https://web.expasy.org/protparam/, accessed on 1 July 2024). Using TBtools software (Ver. 2.142) with default parameters, the distribution of intron-exons and conserved domains of LsIAAs were visualized. The conserved domains of proteins were analyzed by NCBI CD-search (https://www.ncbi.nlm.nih.gov/cdd/, accessed on 1 July 2024).

### 4.4. Chromosome Localization and Synteny Analysis of LsIAA Genes

Gene annotation files and genome files for lettuce were obtained in Phytozome. Chromosome localizations were visualized in TBtools using default parameters [42]. Using the one-step Mcscanx function of TBtools, the intra-species collinearity of *LsIAAs* was analyzed. Subsequently, we downloaded gene annotation files and gene files for *Arabidopsis* and rice on TAIR and Phytozome, respectively. Whole genome collinearity analysis of lettuce, and collinearity analysis of lettuce with *Arabidopsis* and rice were analyzed by TBtools [43].

### 4.5. Analysis of Cis-Acting Elements of LsIAA Genes

The obtained sequences were uploaded to the online website PlantCare (https://bioinformatics.psb.ugent.be/webtools/plantcare/html/, accessed on 1 July 2024) to predict the promoter region of the cis-acting elements, and the promoter cis-acting elements were mapped using TBtools software. By further screening, some important cis-acting elements were screened and the number of individual cis-acting elements per gene was visualized. Subsequently, we used the PLACE database (http://www.dna.affrc.go.jp/htdocs/PLACE/, accessed on 1 July 2024) to verify the results of cis-acting elements obtained from PlantCare, and the results were generally consistent.

### 4.6. Tissue-Specific Expression of LsIAA Genes

The lettuce RNA-Seq data (GSE143675) were downloaded from the Gene Expression Omnibus (GEO) (https://www.ncbi.nlm.nih.gov/geo/, accessed on 1 July 2024) in the NCBI. The expression patterns of *LsIAAs* in different organs (root, stem, leaf, flower, and seed) were visualized using the TBtools software [42], and the normalized expressions of *LsIAAs* were analyzed to generate heatmap.

### 4.7. Plant Material and Treatments

The experiments were conducted in a greenhouse at the Beijing Vegetable Research Center, Beijing Academy of Agricultural and Forestry Sciences. The seeds of lettuce GB-30, which were stored at room temperature in our laboratory, were germinated at 13 °C in the dark. The seedings with uniform size were selected and planted in a matrix of peat and vermiculite (1:1 by volume) under the following conditions: 14/10 h (day/night), 20/13 °C (day/night). While the fourth leaf was fully extended, lettuce plants were transferred to a greenhouse. Plants were subjected to high-temperature conditions (33 °C/25° C, day/night), or regular growth conditions aforementioned. Leaf samples were started on days 0, 2, 8, 16, and 24, and the harvested samples were immediately frozen in liquid nitrogen and stored in a −80 °C refrigerator.

### 4.8. Analysis of Relative Expression of LsIAAs Under High Temperature Treatment

Total RNA was extracted with FastPure Universal Plant Total RNA Isolation Kit (Vazyme, Beijing, China) with modifications [43]. RNA is reverse transcribed into cDNA using the TransScript IV One-Step gDNA Removal and cDNA Synthesis SuperMix (Transgene, Beijing, China). Quantitative RT-PCR (qRT-PCR) was conducted as previously described method [44]. qRT-PCR primers are listed in Supplementary Table S4, and lettuce 18S rRNA (HM047292.1) was used as the reference gene for normalizations.

### 4.9. Subcellular Localization of LsIAA Proteins

To detect the subcellular localization of lettuce IAA proteins, full-length cDNAs of *LsIAA3/4/17/21* were amplified from lettuce. The amplified fragments were cloned into *pCambia1300-GFP* to generate *pCambia1300-LsIAA3-GFP*, *pCambia1300-LsIAA4-GFP*, *pCambia1300-LsIAA17-GFP* or *pCambia1300-LsIAA21-GFP*, respectively. Then, the resulting plasmids were transformed into *A. tumefaciens* (GV3101) and infiltrated into tobacco (*N. benthamiana*) leaves. Plants were incubated in the dark at 24 °C for one day and then transferred to a greenhouse with a photoperiod of 16 h of light/8 h of darkness for another day. Tobacco cells were observed under an orthogonal fluorescence-focusing microscope. Primers of subcellular localization experiments are listed in Supplementary Table S4.

### 4.10. Predicted Protein Interaction Network and Co-Expression Network Construction

The interacting networks of LsIAA and LsARF proteins were integrated with the STRING (https://cn.string-db.org/, accessed on 1 July 2024), and the co-expression network data were exported from STRING [45].

## 5. Conclusions

Our study provides a comprehensive analysis of the Aux/IAA gene family in lettuce, highlighting the functional diversity and evolutionary stability of this important class of proteins. The *LsIAA* genes were expressed in all organs detected, whereas some genes showed tissue-specific expression. Cis-acting elements in response to phytohormones were enriched in the promoters of *LsIAA*s, whereas stress-responsive elements, such as TCA-motif, LTR, and AREB, were found in their promoter regions. qRT-PCR experiments showed that the expressions of most *LsIAA* genes were regulated by high temperatures, indicating that LsIAAs might be potential mediators in heat stress tolerance. Therefore, this study provides valuable information for further investigation of the biological functions of *LsIAA* genes in high-temperature mechanisms. Additionally, the findings from *LsIAA*s will provide candidate targets for the development of new cultivars that are more tolerant to high temperatures.

## Figures and Tables

**Table 1:** Physical and chemical properties of LsIAAs.

Gene Name	Locus ID	Length (aa)	MW (kDa)	pI	II	AI
*LsIAA1*	Lsat_1_v5_gn_1_34161	197	22.14	5.54	46.96	74.06
*LsIAA2*	Lsat_1_v5_gn_1_35400	320	35.45	5.32	45.27	74.06
*LsIAA3*	Lsat_1_v5_gn_1_75561	157	17.78	9.36	30.74	70.70
*LsIAA4*	Lsat_1_v5_gn_3_62041	198	22.74	4.83	34.62	85
*LsIAA5*	Lsat_1_v5_gn_3_84401	288	31.35	5.54	50.37	71.32
*LsIAA6*	Lsat_1_v5_gn_3_125741	234	25.90	5.97	36.29	68.76
*LsIAA7*	Lsat_1_v5_gn_4_98481	278	31.44	5.71	49.29	75.29
*LsIAA8*	Lsat_1_v5_gn_4_101421	310	33.69	8.12	59.33	72.65
*LsIAA9*	Lsat_1_v5_gn_4_104420	146	15.80	5.09	39.02	73.36
*LsIAA10*	Lsat_1_v5_gn_4_104440	189	21.46	5.31	50.74	78.84
*LsIAA11*	Lsat_1_v5_gn_4_157881	340	36.64	7.54	47.54	69.41
*LsIAA12*	Lsat_1_v5_gn_5_29420	179	20.96	4.74	51.25	66.37
*LsIAA13*	Lsat_1_v5_gn_5_66981	222	24.47	8.25	38.94	64.14
*LsIAA14*	Lsat_1_v5_gn_5_67581	188	21.20	5.98	49.68	56.54
*LsIAA15*	Lsat_1_v5_gn_5_81461	317	35.05	4.64	54.70	73.75
*LsIAA16*	Lsat_1_v5_gn_5_85421	168	19.03	6.79	62.37	95.12
*LsIAA17*	Lsat_1_v5_gn_5_129861	241	27.13	5.86	39.00	82.90
*LsIAA18*	Lsat_1_v5_gn_5_134641	309	33.88	7.52	48.86	65.31
*LsIAA19*	Lsat_1_v5_gn_5_186760	181	20.50	5.60	56.23	64.59
*LsIAA20*	Lsat_1_v5_gn_6_46381	97	11.00	4.05	55.43	72.37
*LsIAA21*	Lsat_1_v5_gn_7_41401	287	31.35	8.52	40.64	68.92
*LsIAA22*	Lsat_1_v5_gn_7_44161	212	23.72	5.12	43.02	63.4
*LsIAA23*	Lsat_1_v5_gn_8_62421	213	23.71	6.08	43.44	74.55
*LsIAA24*	Lsat_1_v5_gn_8_91000	261	27.70	5.04	46.17	76.17
*LsIAA25*	Lsat_1_v5_gn_8_91920	181	20.50	6.62	48.97	67.79
*LsIAA26*	Lsat_1_v5_gn_8_95700	298	32.58	8.06	47.85	71.64
*LsIAA27*	Lsat_1_v5_gn_9_10001	238	26.18	5.73	39.72	63.87
*LsIAA28*	Lsat_1_v5_gn_9_10020	181	20.42	5.36	53.99	66.69
*LsIAA29*	Lsat_1_v5_gn_9_11520	274	29.14	5.80	46.63	75

## Data Availability

The original contributions presented in this study are included in the article/Supplementary Materials. Further inquiries can be directed to the corresponding author(s).

## Supplementary Materials

The following supporting information can be downloaded at: https://www.mdpi.com/article/10.3390/ijms26041687/s1.
